# Simulating an emergency department: the importance of modeling the interactions between physicians and delegates in a discrete event simulation

**DOI:** 10.1186/1472-6947-13-59

**Published:** 2013-05-22

**Authors:** Morgan E Lim, Andrew Worster, Ron Goeree, Jean-Éric Tarride

**Affiliations:** 1 Department of Clinical Epidemiology and Biostatistics, McMaster University, Hamilton, ON, Canada; 2 Programs for Assessment of Technology in Health (PATH) Research Institute, St. Joseph’s Healthcare Hamilton, 25 Main St. W., Suite 2000, Hamilton, ON, L8P 1H1, Canada; 3 Department of Medicine, Division of Emergency Medicine, McMaster University, Ontario, Canada

## Abstract

**Background:**

Computer simulation studies of the emergency department (ED) are often patient driven and consider the physician as a human resource whose primary activity is interacting directly with the patient. In many EDs, physicians supervise delegates such as residents, physician assistants and nurse practitioners each with different skill sets and levels of independence. The purpose of this study is to present an alternative approach where physicians and their delegates in the ED are modeled as interacting pseudo-agents in a discrete event simulation (DES) and to compare it with the traditional approach ignoring such interactions.

**Methods:**

The new approach models a hierarchy of heterogeneous interacting pseudo-agents in a DES, where pseudo-agents are entities with embedded decision logic. The pseudo-agents represent a physician and delegate, where the physician plays a senior role to the delegate (i.e. treats high acuity patients and acts as a consult for the delegate). A simple model without the complexity of the ED is first created in order to validate the building blocks (programming) used to create the pseudo-agents and their interaction (i.e. consultation). Following validation, the new approach is implemented in an ED model using data from an Ontario hospital. Outputs from this model are compared with outputs from the ED model without the interacting pseudo-agents. They are compared based on physician and delegate utilization, patient waiting time for treatment, and average length of stay. Additionally, we conduct sensitivity analyses on key parameters in the model.

**Results:**

In the hospital ED model, comparisons between the approach with interaction and without showed physician utilization increase from 23% to 41% and delegate utilization increase from 56% to 71%. Results show statistically significant mean time differences for low acuity patients between models. Interaction time between physician and delegate results in increased ED length of stay and longer waits for beds.

**Conclusion:**

This example shows the importance of accurately modeling physician relationships and the roles in which they treat patients. Neglecting these relationships could lead to inefficient resource allocation due to inaccurate estimates of physician and delegate time spent on patient related activities and length of stay.

## Background

Overcrowded emergency departments (ED) are an ongoing issue for hospital staff, healthcare administrators, policy makers and patients. With increasing patient demands on these services and constricting budgets, administrators are in search of practical and implementable solutions (e.g. staff scheduling and resource allocation) to minimize patient waiting time and increase throughput. Methods of computer simulation have often been employed to model ED activity because it allows researchers to analyze the effects of re-organizing resources in the ED without making potentially costly changes.

A review of 29 studies identified four mathematical modeling techniques used to evaluate waiting times in the ED [1]: analytic queuing models (n=3), system dynamics (n=2), discrete event simulation (DES) (n=22), and agent-based modeling (ABM) (n=2). DES was the most frequently (75%) used modeling technique. Compared to analytic queuing models and system dynamics, DES is capable of modeling more complex non-linear systems while taking into account patient history, staff scheduling and multiple resource constraints. It is a process-oriented model that is represented by a network of queues for services that a patient flows through where attributes determine the pathway of the patient. The drawback is that modelers continue to view the hospital like a factory where the patient is the driver [2]. In a DES model, patients queue, are triaged and wait for a resource (e.g. bed, physician) based on their acuity levels (e.g. high priority patients are served first). Once that resource (e.g. physician) has completed processing (e.g. treating patient) it immediately moves onto the next patient. This is an unrealistic depiction of ED care because physicians have a skill hierarchy where a physician will most likely not perform a task that can easily be performed by a delegate such as a medical student, resident physician, physician assistant, or nurse practitioner. A number of studies have argued for the inclusion of skill-based specification which would allow a physician or delegate to prioritize tasks and produce a more realistic result [2,3].

Process-oriented models also tend to neglect indirect patient-related tasks that physicians are required to perform (e.g. teaching, consultation with a specialist, charting, and discussions with the patient’s family). In the review [1], only one previous DES study attempted to include multi-tasking by fragmenting physicians and nurses into several parts where each part represented a task [4]. Consequently, this study did not incorporate interaction between the physician and their delegates, which is a common limitation in previous simulation studies of the ED.

These indirect patient-related tasks play an even larger role when modeling a teaching hospital because a large portion of the ED staff is comprised of physician trainees. The physician trainee’s function is to both treat patients and learn from senior staff. Despite these differences, the interactions between trainees and physicians are frequently neglected. If included, trainees are often modeled similarly to physicians where the only difference exists in the assessment/treatment time [4-7]. However, previous research has found that the presence of trainees in the ED is positively associated with an increased patient length of stay (LOS) [8,9] and that trainees exhibit poor time management when faced with overcrowding [10,11]. Time and motion studies [12,13] have estimated that approximately 30% of a physician’s time is actually spent with the patient (e.g. examining and treating) while the remainder is spent on other tasks such as teaching, charting, interactions with the nursing staff and addressing family member concerns. As such, ignoring indirect patient demands by ignoring the interactions between physicians and their delegates may result in an overestimation of staff resource availability and thus provide inaccurate estimates of resource utilization (percent of scheduled time spent with patient) and patient LOS.

To overcome some of these issues, analysts have used agent-based modeling (ABM). Agents can represent people (e.g. patients, physicians), services (e.g. diagnostics) and the environment in which they operate and are governed by a set of goals (e.g. minimize time spent with patient) and behaviours (e.g. treat). Agents are able to make autonomous decisions, interact with each other and exhibit proactive behaviour based on their internal goals [14]. As such, the purpose of ABM models is to model at the individual level and observe the emergent behaviour and detailed movement pattern. The application of ABM to evaluate healthcare problems is still relatively new compared to the use of DES. Previous work has explored the use of ABM to model different ED physician staffing schedules [15], patient diversion strategies [16], and differing radiology process times [17]. Only one model identified accounting for differing levels of staff expertise [18], however, as indicated by the authors, this model is still in its first cycle of development.

ABMs are also difficult to implement because these types of models are often based on theories or subjective data (e.g. expert opinion) and few user friendly software exist for enthusiasts who are not expert programmers [14]. Nonetheless, an agent’s deliberative process is inherently discrete and using an event-based approach (as opposed to discrete time) eases the integration of agents interacting (when they should), which may be more representative of reality [19]. When modeling the ED, pure ABMs may be limited because there is no concept of queues and flows and would need to be combined with DES [14].

To overcome these issues, a new method was developed to model a hierarchy of heterogeneous interacting pseudo-agents in a DES, where pseudo-agents are entities with embedded decision logic [20]. The purpose of this study is to present an alternative approach where physicians and their delegates in the ED are modeled as interacting pseudo-agents in a DES and to compare it with the traditional approach ignoring such interactions. To the best of our knowledge, this approach of using interacting pseudo-agents has never been implemented when modeling the ED using DES.

## Methods

This new approach of modeling physicians and their delegates as interacting pseudo-agents is first validated before being implemented in an ED DES model. Results are compared to the traditional approach without interaction. The following provides a description of the interacting pseudo-agents approach and the models used to compare this new approach with the traditional way of modeling the ED without interactions between physicians and their delegates. The models are built using the Arena® Simulation Software, version 13.9 (Rockwell Automation).

### Development of the interacting pseudo-agents approach

Physicians and delegates are considered pseudo-agents because they are modeled as entities with embedded hierarchical decision-logic as opposed to being true autonomous agents found in pure ABM. To model interactions, separate entities are created for the physician and delegate. Each entity is assigned a new set of resource states: idle (waiting for a patient), assessing patient (only for delegate to develop treatment plan), consulting (with each other), or treating patient. The interaction occurs once the delegate has assessed a patient and requires consultation with the physician about the treatment plan. The model assumes that at anytime one physician and one delegate are scheduled. Decisions made by the physician and delegate are summarized below.

1. Physician: There is one attending physician in this model per shift. The role of the physician is to treat patients and produce orders (i.e. laboratory work, xray, discharge/admit) which dictate the pathway of the patient. Their other role is to aid delegates with their patient assessments. The physician attends to patients of a higher priority before anything else. If there are no high priority patients in queue for treatment then the physician will attend to any delegates who are waiting for guidance with their patient assessments. If there are no high priority patients or delegates who need assistance then they will treat a low priority patient.

2. Delegate: There is one delegate in this model. Their role is to assess and treat patients with a low priority. Once they have developed a treatment plan they must check with the physician before proceeding to treatment.

**Figure 1:**
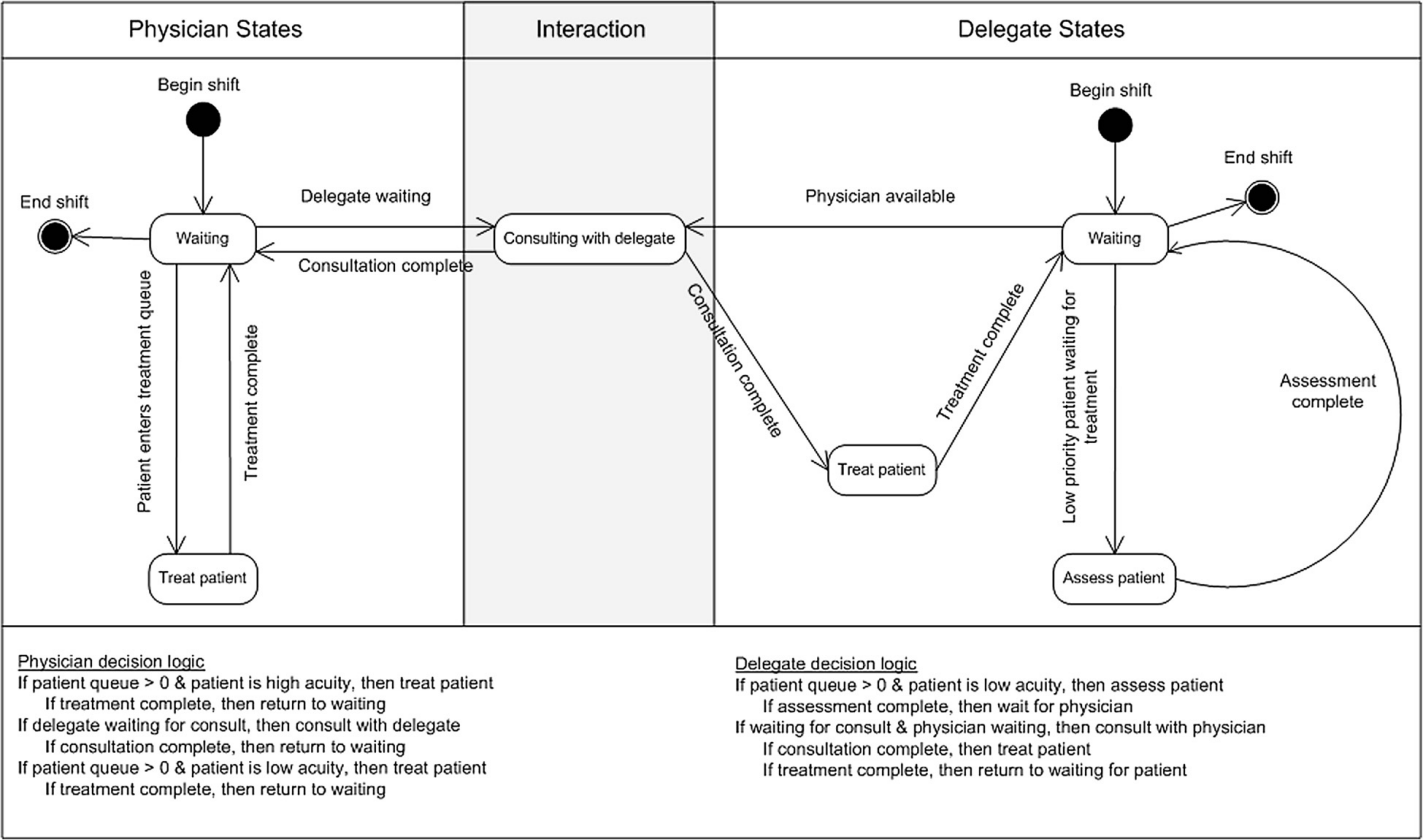
Possible physician and delegate states and state transitions.

Figure 1 represents the states and state transitions of the physician and delegate. In this approach there is one queue for patients. The physician and delegate entities are held in a hold block until a signal is sent to release them. There are two signals: one is sent to both the physician and delegate to alert that there is a patient waiting in the queue and the other is sent to the physician from the delegate to alert that the delegate is waiting for a consult.

### Validation of the interacting pseudo-agents approach

A simple model without the complexity of the ED is created in order to validate the building blocks (programming) used to create the pseudo-agents and their interactions. Using a simple model controls for any differences that might arise as a result of system feedback as opposed to the programming. The objective of the validation exercise is to compare the outputs from the same model built using first the conventional blocks (i.e. physician as a resource with no interactions with delegates) and secondly the new building blocks modeling the physician and delegate as entities with interaction. If the blocks representing the interaction are disabled, then the results should be similar with the model built using the conventional blocks (i.e. no interactions). This should hold true because, in both models, the physician will treat all high priority before low priority patients and delegates will only treat low priority patients. Once the interaction blocks are enabled, there should be an increase in resource utilization and waiting time for low priority patients because the physician has to consult with the delegate about the patient’s treatment plan before proceeding.

The following assumptions are used for the model developed to validate this new method. Patients enter the model and then queue for treatment. Once treatment is complete the patient is discharged from the model. Approximately 5 patients arrive per hour (20% high acuity and 80% low acuity) with an exponentially distributed inter-arrival rate. The only processing time is related to treatment time which is assumed to be 20 minutes for high acuity patients and 10 minutes for low acuity patients. An additional 10 minutes is added to the treatment time when the patient is processed by a delegate. In the interacting pseudo-agent approach it is assumed that delegates spend 5 minutes interacting with the physician before returning to the patient. An additional 5 minutes is added to implement any instructions by the physician. All simulations are run for a 24 hour warm-up period and then for 24 hours over 500 replications.

Resource utilization (percentage of scheduled time spent on patient related activities which includes consultation for the interacting pseudo-agent approach) and time to disposition based on acuity and whether treated by a physician or delegate were compared for three sets of outputs: 1) using the conventional building blocks, 2) using the new building blocks without the interaction between physician and the delegate and, 3) using the new building blocks with the interaction between physician and the delegate. The assumption behind the validation exercise is that comparisons (1) and (2) should be comparable in terms of percentage of time spent on patient related activities and that differences should exist with outputs from (3).

### Implementation of the interacting pseudo-agents approach in a hospital emergency department

Once validated, the interacting pseudo-agent approach is implemented in a more realistic representation of a DES model of a hospital ED. The following presents the assumptions of the ED model which is informed by data from an academic hospital from Hamilton, Ontario, Canada.

#### Process overview

**Figure 2:**
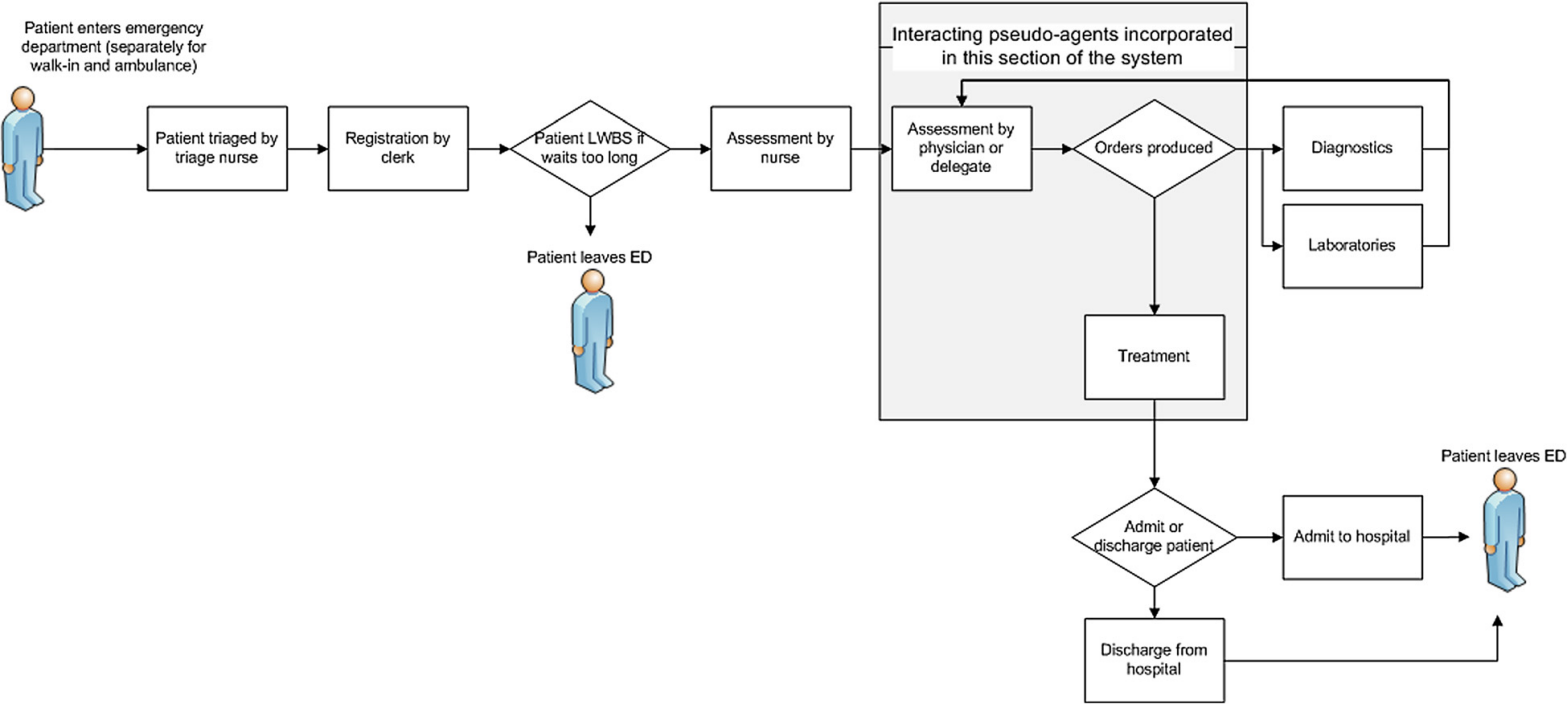
Hospital emergency department patient flow.

The ED is open 24 hours and is responsible for triage and treatment of approximately 50,000 patients a year. Figure 2 presents the basic patient flow through the ED and the point in the patient flow where the interacting pseudo-agents approach is incorporated into the model. The patient arrives as a walk-in or by ambulance and proceeds to triage. The patient is triaged by the triage nurse and then registered by the clerk. In this hospital, the patient can only be admitted to the ED when both the charge nurse and a bed are available. If both are unavailable the patient is seated in the waiting room and enters a queue. In some cases, a patient may voluntarily leave the ED without being seen after a prolonged wait. However, for the vast majority of patients, the patient is placed in a bed and assessed by the nurse when they become available. Once the nurse assessment is complete, the patient is placed in queue to see the physician or the delegate depending on the patient’s acuity. When available, the physician assesses the patient and produces orders: 1) send for diagnostics, 2) send for laboratories (i.e. blood work) or 3) treat. If a laboratory is ordered the patient will wait in bed until a bedside nurse is available to draw blood. The patient does not have to wait for results before proceeding. If radiology is ordered the patient is sent to the radiology room which is located in the ED. After radiology the patient returns to the same bed. After treatment the patient is either admitted to the hospital or discharged from the ED.

#### Entities

There are three types of entities in this model: 1) patients, 2) physician, and 3) delegate. The physician and delegate are modeled as entities only in the interacting pseudo-agent approach. Patients are triaged into one of five categories according to the Canadian Triage and Acuity Scale (CTAS) where level one is the most severe: level 1 (resuscitation), level 2 (emergent), level 3 (urgent), level 4 (less urgent) and level 5 (non-urgent) [21]. Because CTAS 1 and 2 patients are treated similarly in the model, results are reported as high (CTAS 1 and 2) and low (CTAS 3, 4, and 5) acuity. The physician treats all patients with priority given to CTAS 1 and 2 and the delegate only treats low acuity patients.

**Figure 3:**
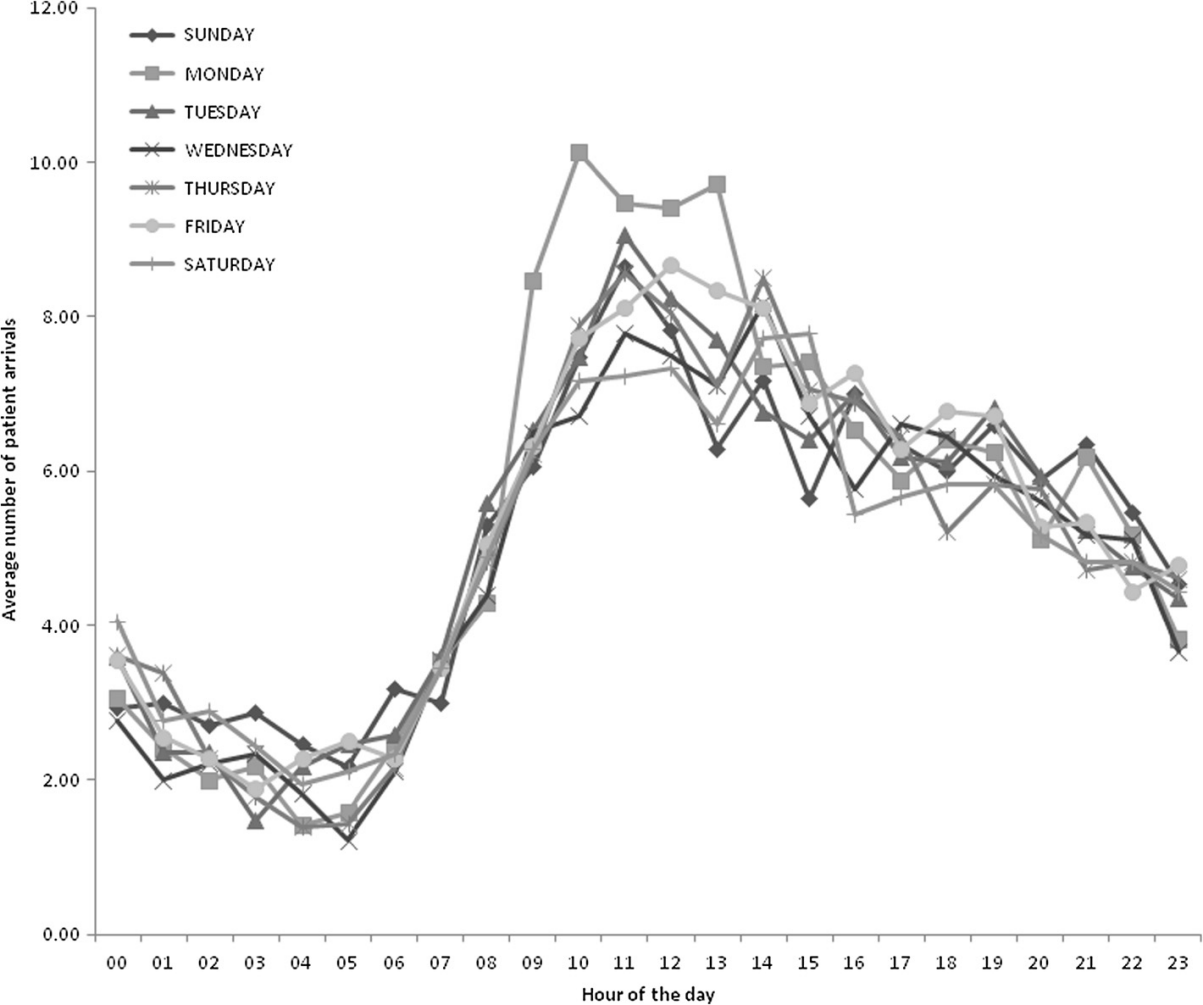
Weekday patient arrival pattern over a 24 hour period.

#### Input data

Patient arrival times and probabilities used in the hospital ED model are derived from a hospital administrative database with information on each patient that arrives in the ED. Patient arrival is based on a schedule derived from the data (separate for walk-in and ambulance) that changes hourly and daily to account for peak and non-peak times. Figure 3 presents the average number of patient arrivals by weekday and hour. An exponential distribution is used to distribute the arrivals over each hour. Data and time stamps are entered manually into a centralized database by ED staff (e.g. clerks, nurses, and physicians). Data is used from 15,196 patients collected between April and July 2010. Process times are derived from a time and motion study (n=85) conducted in the ED over a 24 hour period. Table 1 and Table 2 summarize the inputs as well as the probability distributions used in the model to reflect the variation associated with the mean values. In Table 1, triage nurse and registrar capacity is based on an hourly schedule. In Table 2, the probabilities of having blood work or being sent to radiology is dependent on CTAS level but are independent of each other. Distributions are derived using Arena’s input analyzer, which fits probability distribution functions to the data.

**Table 1:** Input data

**Resource**	**Capacity†**	**Process time (minutes)**	**Distribution**
Triage nurse	1 or 2	10	Poisson
Registrar	1 or 2	2	Lognormal
Bedside nurse	2		
*Assessment*		10	Beta
*Draw blood*		4	Triangular
*Discharge*		10	Triangular
Charge nurse	1		
Physician	1		
Treatment (*high priority*)		9	Triangular
Treatment (*low priority*)		4	Triangular
*Consultation*		4.5	Triangular
Delegate	1		
Treatment (*low priority*)		4	Triangular
*Consultation*		4.5	Triangular
Beds	5	NA	
Radiology‡	1	9	Beta

All inputs were estimated based on the time and motion study except radiology.

†Based on schedule, ‡ Estimated from administrative data.

**Table 2:** Probability inputs to the model

	**All**	**CTAS 1**	**CTAS 2**	**CTAS 3**	**CTAS 4**	**CTAS 5**
Probability of patient with CTAS level		0.01	0.16	0.56	0.25	0.02
Probability of patient receiving radiology		0.82	0.07	0.48	0.28	0.18
Probability of patient having blood work		0.85	0.73	0.51	0.19	0.01
Probability of leaving without being seen						
< 30 minutes	0.01					
30-60 minutes	0.02					
60-120 minutes	0.1					
120-180 minutes	0.2					
180-300 minutes	0.3					
> 300 minutes	0.4					

Inputs estimated from administrative data.

#### Initialization and analysis

To avoid initialization bias, a warm-up period was chosen using Welch’s method [22]. The model is simulated over multiple replications for an extended period where key performance measures are recorded. The moving averages of these measures are graphed over time. Once the average stabilizes there is no longer initialization bias and the model reaches a steady state. This is considered the warm-up period and statistics begin recording after this time point. Analysis of simulation outputs is performed using the batch means method, where only one simulation run is executed. Data accrued during the warm-up period are deleted and the remainder of the run is divided into batches and each batch average represents a single observation. Arena has a built-in batching algorithm [23] that performs the analysis and provides a batched mean average with 95% confidence intervals (CI) in a report.

### Comparison of outputs between the interacting pseudo-agent approach and the approach without interaction

The purpose of this comparison is to evaluate the performance of the approach with interacting pseudo-agents compared to the approach without interaction in terms of resource utilization, patient waiting time for assessment and treatment and flow time. Resource utilization is defined as the percentage of scheduled time spent on patient-related activities which includes assessment, treatment and interaction time (i.e. consultation). The following time intervals are estimated for the patient and reported by acuity level and whether treated by physician or delegate: arrival to bed (includes waiting time for triage and bed), waiting time for physician or delegate based on acuity, process time for assessment, treatment, and consultation and average total LOS. Model outputs are compared with two-sample t-tests using Arena’s output analyzer. To better understand the effect of various changes on the operation of the ED system, sensitivity analyses are conducted on several key parameters including patient demand, number of beds and nurses, and consultation time.

## Results

The following reports on the validation of the interacting pseudo-agents approach and the comparison of this approach and the approach without interaction when implemented in a hospital ED informed by real data.

### Validation results

Table 3 presents the results of the validation of the interacting pseudo-agent approach implemented in a simple model. As expected, physician and delegate utilization (64% and 72% respectively) is similar between the approach without interaction and the interacting pseudo-agent approach when the interaction between physician and delegate is disabled. Likewise, time to disposition for low and high acuity patients are identical between the two approaches. Once the interaction between physician and delegate is enabled in the pseudo-agent approach, there is an increase in resource utilization for the physician and delegate (25% and 21% respectively). The time to disposition is similar in high acuity patients because they are first priority over teaching. In contrast, low acuity patients are processed slower because they must wait in queue while the physician processes high acuity patients or teaches the delegate. When seen by a delegate low acuity patients are processed the slowest because extra time is taken for the delegate to go over the treatment plan with the physician and then to administer said treatment.

### Comparison of outputs between the interacting pseudo-agent approach and the approach without interaction

Results from the warm-up analysis showed average output stabilization after 40 days. The model was run for one replication of 420 days which was long enough to provide enough observations to calculate 95% CIs using the batched means methods. Table 4 reports outputs comparing the interacting pseudo-agents approach and the approach without interaction when implemented in a hospital ED model. Similar to results in Table 3, the utilization increases when the interaction between physician and delegate is incorporated. When compared to the model without interaction, physician utilization increases by approximately 78% (from 23% to 41%) while delegate time increases by 27% (from 56% to 71%). Additionally, in the model with interaction, the physician remains idle (i.e. waiting to perform a task) on average 7 minutes before seeing a patient, whereas the delegate remains idle 10 minutes on average. A short waiting time is estimated for patients waiting for assessment and treatment from the physician or delegate. Despite this short waiting time, they are still statistically different between approaches, implying that patients do wait longer when the physician and delegate are assigned other patient related tasks (i.e. consultation). Patient time spent with the physician is similar between models because the treatment time input remains the same, however, patient time with the delegate increases (14.5 minutes to 24.5 minutes) because the delegate must consult with the physician before proceeding with treatment. In turn, this increases LOS which results in a longer queue for beds. Statistically significant differences are observed for average LOS between the two approaches for all low acuity patients, however, a statistically significant difference is not observed for high acuity patients. This is most likely due to the variation between time to admission and time to discharge.

### Sensitivity analysis

Sensitivity analysis is conducted on several parameters: the patient walk-in arrival rate is decreased by half, consultation time between the physician and delegate is increased, time to treat the patient is increased and the number of resources is increased (beds, charge nurses, and bedside nurses). Table 5 reports on the results of the sensitivity analysis. Physician and delegate utilization increases when consultation time is increased but remains the same when resources are increased. Time from arrival to bed for high acuity patients does not vary significantly for all analyses, however, low acuity patients are placed in a bed quicker when the number of beds and bedside nurses is increased. As a result, these resource changes also lead to a decrease in average LOS. This may indicate that the number of beds and bedside nurses are limiting the flow of patients through the ED model. Increasing consultation time (i.e. interaction time) increases patient waiting time for the physician (4.49 for high acuity and 5.28 minutes for low acuity) and delegate (3.31 minutes). This translates into a longer time from arrival to bed for low acuity patients because it takes longer to discharge patients.

**Table 3:** Validation results of the interacting pseudo-agent approach

**Performance measure**	**Without interaction**	**Pseudo-agent**	**Pseudo-agent**
		**(interaction disabled)**	**(interaction enabled)**
Utilization*			
Physician	0.64 (0.63, 0.65)	0.64 (0.63, 0.65)	0.89 (0.88, 0.90)
Delegate	0.72 (0.72, 0.72)	0.72 (0.72, 0.72)	0.93 (0.93, 0.93)
Time to disposition (minutes)			
Physician (High acuity)	26.97 (26.68, 27.26)	26.97 (26.68, 27.26)	28.42 (28.13, 28.71)
Physician (Low acuity)	18.92 (18.41, 19.43)	18.92 (18.41, 19.43)	83.97 (78.37, 98.57)
Delegate (Low acuity)	28.13 (27.61, 28.65)	28.13 (27.61, 28.65)	110.94 (105.15, 116.73)

*time for teaching or learning only included in pseudo-agent model (95% confidence intervals).

**Table 4:** Results comparing two approaches using the hospital emergency department model

**Performance measure**	**Value (95% CI)**	**Difference (SD)**
Utilization**		
Physician-Without interaction	23%	18%
With reaction	41%	
Delegate-Without interaction	56%	15%
With interaction	71%	
Time from arrival to bed (minutes)		
High acuity-Without interaction	20.39 (20.06, 20.72)	1.08* (0.26)
With interaction	21.47 (21.19, 21.75)	
Low acuity-Without interaction	68.94 (62.87, 75.01)	19.55* (1.47)
With interaction	88.49 (80.36, 96.62)	
Patient waiting time for physician or delegate (minutes)		
Physician (high acuity)-Without interaction	0.49 (0.44, 0.54)	0.53* (0.04)
With interaction	1.02 (0.96, 1.08)	
Physician (low acuity)-Without interaction	0.56 (0.55, 0.57)	0.93* (0.02)
With interaction	1.49 (1.46, 1.52)	
Delegate (low acuity)-Without interaction	0.15 (0.14, 0.16)	0.37* (0.01)
With interaction	0.52 (0.49, 0.55)	
Patient time with the delegate (minutes)		
Delegate (low acuity)-Without interaction	14.50 (14.5, 14.5)	10.01* (0.01)
With interaction	24.51 (24.48, 24.51)	
Average total length of stay (minutes)		
Physician (high acuity)-Without interaction	85.91 (85.15, 86.67)	0.92 (0.61)
With interaction	86.83 (85.88, 87.78)	
Physician (low acuity)-Without interaction	118.54 (111.13, 125.78)	18.45* (1.31)
With interaction	136.99 (128.47, 145.51)	
Delegate (low acuity)-Without interaction	114.84 (108.32, 121.36)	27.23* (1.36)
With interaction	142.07 (135.48, 148.66)	
All low acuity patients-Without interaction	116.66 (110.49, 122.83)	23.1* (0.94)
With interaction	138.91 (130.86, 146.96)	

*statistically significant at the 5% level, **includes interaction time.

*CI* confidence interval, *SD* standard deviation.

## Discussion/Conclusion

The ED is a complex environment that involves a number of interactions between patients and staff. Relationships between physicians and their delegates are also an important part of the overall ED process, perhaps more so in teaching hospitals. The objective of this study is to introduce an approach to modeling physicians that incorporates both embedded decision logic and physician interaction when modeling the ED. This interacting pseudo-agent approach is first validated using a simple model before being implemented in a more comprehensive hospital ED model informed by data from a Canadian academic hospital. Comparison between the model with interaction and the model without interaction showed that resources are less idle and lower acuity patients are processed slower because of consultation time. These results are corroborated by the literature that show decreased throughput when delegates are working. In the ED model, physician utilization increases from 23% of scheduled time to 41% when the interaction is modeled. This physician utilization is similar to those found in two separate time and motion studies documenting emergency physician time utilization. Friedman et al [24] observe that direct patient related physician utilization is 28% and that teaching medical students and residents accounts for 5.8%. Hollingsworth et al [13] found 32% of physician utilization was related to direct patient related activities and 6.3% was related to teaching, however, percentage of time spent on teaching is most likely an underestimate as only interactions with senior residents was recorded which would bring it closer to our results.

**Table 5:** Sensitivity analysis results

**Parameter**	**Utilization**		**Time from arrival to bed (minutes)**		**Average total length of stay (minutes)**		
	**Physician**	**Delegate**	**High acuity**	**Low acuity**	**Physician (High Acuity)**	**Physician (Low Acuity)**	**Delegate (Low Acuity)**
Base case	41%	71%	21.47 (21.19, 21.75)	88.49 (80.36, 96.62)	86.83 (85.88, 87.78)	136.99 (128.47, 145.51)	142.07 (135.48, 148.66)
Patient walk-in arrival (1/2)	26%	57%	15.84 (15.61, 16.07)	18.99 (18.50, 19.48)	77.39 (76.12, 78.66)	58.25 (57.39, 59.11)	74.33 (73.74, 92)
Number of beds (from 5 to 10)	41%	70%	17.60 (17.13, 18.07)	31.06 (28.53, 33.59)	92.92 (90.70, 95.14)	89.68 (84.69, 94.67)	100.76 (96.96, 104.56)
Number charge nurses (from 1 to 2)	41%	70%	19.99 (19.08,20.90)	71.68 (55.45, 87.91)	83.56 (80.51, 86.61)	117.98 (101.96, 134)	127.13 (106.55, 147.71)
Number of bedside nurses (from 2 to 3)	41%	70%	18.79 (18.46, 19.12)	39.88 (37.25, 42.51)	78.91 (77.87, 79.95)	78.79 (75.76, 81.82)	91.73 (89.36, 94.10)
Consultation time (5 to 15 minutes)	66%	86%	22.65 (22.65, 23.05)	130.32 (111.79, 148.85)	89.71 (88.76, 90.66)	179.36 (161.17, 197.55)	197.56 (177.78, 217.34)

(95% confidence intervals).

Using an interacting pseudo-agents based approach in DES can be particularly beneficial if the purpose of the simulation model is to derive optimal staff scheduling as this approach provides a more accurate representation of scheduled resource utilization and patient throughput. It is also possible to extend this method to other delegates (e.g. nurse practitioners, medical students, interns and physician assistants) to incorporate breaks and shift length for scheduling or to model more complex decision making to determine bottlenecks in patient flow. In the ED setting, the role of physician delegates is to assist with patient treatment under the supervision or in consultation with the physician. As such, interaction with physician delegates consumes a significant amount of the physician’s time which needs to be modeled.

If time and resources are available, a full ABM model can be built rather than embedding decision logic in entities in a DES. This method models patients, physicians and nurses as separate agents where each operate under a different set of rules and interact more like a real ED. The drawback is that an ABM model takes longer to build as it is more complex and requires more data and knowledge of decision rules than other simulation techniques. Although the use of ABM in healthcare is gaining in popularity, it remains limited by the unavailability of commercial software and analysts with expertise in ABM. This may explain why DES is more commonly chosen to model the ED. The International Society for Pharmacoeconomics and Outcomes Research and the Society for Medical Decision Making Modeling Good Research Practices Task Force [20] advocates using DES to model more complex interactions between individuals which can be extended to modeling interactions between physicians and delegates in this study.

Our results show that it is important to model these types of interactions when simulating EDs. Despite its strengths, a few limitations are associated with this study. Both junior and senior delegates are not modeled as we assume that senior delegates function as an attending physician. This assumption is corroborated by an attending emergency physician. In reality, senior delegates are capable of treating patients of all acuity levels with minimal supervision and they also spend a portion of their time teaching junior delegates. Although our model reflects agent decisions that occur in an academic institution, we acknowledge that this may differ between hospitals. Results must also be used with caution as the hospital ED model does not incorporate the entire patient flow or possible variations in routing (e.g. multiple diagnostic tests and re-assessments) due to data limitations. Excluding re-assessment may result in an underestimate of total LOS and resource utilization. Some complexities of the ED are also not included in the model such as high acuity patients by-passing triage and jumping queue for a bed. However, our sensitivity analysis showed that high acuity patients are not greatly affected in the model when changes are made. Additionally, only one delegate shift was illustrated, whereas it is possible for multiple delegates to be working the same shift. Increasing the number of delegates would most likely also increase patient LOS. It is also worth noting that inputs estimated using the time and motion study was based on a small number of observations (n=85). Finally, since the objective of this study is to develop and validate a new modeling approach before comparing it in a hospital ED, we did not focus on comparing the outputs of the ED model with the observed data. Rather we aimed to show that simulation studies which did not model interactions between physicians and delegates may be misleading.

Implementation using commercially available software may not be as desirable as coding a program oneself, however, it presents a viable option for analysts unfamiliar with coding. In terms of scalability, modeling resources as interacting pseudo-agents in a DES may become overly complicated if the objective is to assess interactions among a large variety of staff. Despite these limitations, this study presents for the first time, an approach to model interactions between physicians and delegates and results indicates important differences in resource use and time intervals when compared to methods not modeling such types of interactions.

Future work would be to increase the complexity of the model to incorporate re-assessments and multiple diagnostic pathways. It would also be informative to create interacting pseudo-agents for nurses and residents, medical students and interns. Nurses and physicians spend a significant amount of time consulting about the patient as the nurse is responsible for monitoring patient vitals. Residents, medical students and interns require differing amounts of the attending physician’s time which detracts from the patient, therefore it would be important to incorporate these staff members in the model to more precisely measure physician utilization.

In conclusion, the pseudo-agent based approach provides a more realistic representation of the emergency department. Our results indicate that modeling the interaction between physician and delegate can have an impact on predicted patient throughput and waiting time. In light of these results, future DES modeling of the ED setting should incorporate interaction between physician and delegate by modeling them as pseudo-agents.

## Competing interests

The authors declare no competing interests.

## Authors’ contributions

ML developed the discrete even simulation models, performed the data analysis and drafted the manuscript. AW, RG and JET contributed to the development of the discrete event simulation model, interpretation of the data analysis, and manuscript revisions. All authors read and approved the final manuscript.

## Authors’ information

ML is a PhD Candidate in the Health Research Methodology Program in the Department of Clinical Epidemiology and Biostatistics at McMaster University in Ontario, Canada.

AW is an Associate Professor in the Department of Medicine, Associate Member in the Department of Clinical Epidemiology and Biostatistics at McMaster University and Research Director and Emergency Medicine Physician for Hamilton Health Sciences, Ontario, Canada.

RG is a Professor in the Department of Clinical Epidemiology and Biostatistics at McMaster University and Director at the Programs for Assessment of Technology in Health (PATH) Research Institute, Ontario, Canada.

JET is an Associate Professor in the Department of Clinical Epidemiology and Biostatistics and Associate Member in the Department of Economics at McMaster University.

## Pre-publication history

The pre-publication history for this paper can be accessed here:

http://www.biomedcentral.com/1472-6947/13/59/prepub
